# Human Papillomavirus: Oral Lesions and Vaccination

**DOI:** 10.3390/cancers15102711

**Published:** 2023-05-11

**Authors:** Federica Di Spirito

**Affiliations:** Department of Medicine, Surgery and Dentistry, University of Salerno, 84084 Salerno, Italy; fdispirito@unisa.it or dispiritof@yahoo.it

Human papillomavirus (HPV) is associated with benign and malignant lesions in various locations, such as the skin and oral and genital mucosa [1]. Indeed, HPV is the most important pathogen in anogenital infections, especially of the cervix, while the relationship between anogenital and oral infections seems to be poor and inconsistent [1,2]. Nonetheless, oral HPV infection can occur subclinically or manifest as benign lesions, namely squamous cell papillomas, condyloma acuminata, verruca vulgaris, and focal epithelial hyperplasia [3], as well as potentially malignant lesions, which most notably include proliferative verrucous leukoplakia [4].

Among the approximately 200 HPV genotypes [5], 25 have been associated with oral lesions, including HPV-1, -2, -3, -4, -6, -7, -10, -11, -13, -16, -18, -31, -32, -33, -35, -40, -45, -52, -55, -57, -58, -59, -69, -72, and -73 [6], with the low-risk HPV-6 and -11 being the most frequently detected overall. To be more specific, squamous cell papillomas and condyloma acuminata are commonly associated with HPV-6 and HPV-11; verruca vulgaris are associated with HPV-2 followed by HPV-57, -4, and -40; and HPV-13 and -32 are linked to FEH [6,7].

In addition, anogenital and HPV infection is an independent risk factor for oropharyngeal benign and malignant neoplasms [2]. HPV-related oral squamous cell carcinomas (OSCCs) account for a smaller proportion than oral cancers, which are mostly attributable to other risk factors such as tobacco, alcohol, or areca nut [8]. However, following the decline in tobacco use in several countries, there has been an increase in HPV-associated oral cancers, similar to oropharyngeal cancers, indicating a possible independent role of HPV in OSCCs [9]. Although the role of HPV in the development of OSCCs is still not fully understood [10], HPV approximately represents 3% of oral carcinomas [11], and HPV’s E6 and E7 viral proteins, being able to block two suppressor proteins, are responsible for HPV oncogenic power [12,13]. 

Specifically, E7 binds to p53 by participating in the cleavage of p53 by E6 [14] and causing the loss of control over the G1-S phase transition of the cell cycle, leading, in turn, to an increase in cell cycling [15]. In fact, the altered action of p53 is considered a risk factor for carcinogenesis. Moreover, E7 promotes the degradation of retinoblastoma protein (pRb) and interacts with other cell cycle factors, such as cyclin-dependent kinases (CDK) by inhibiting p27 and p21 [16]. This leads to the dysregulation of the cell cycle. E7 also causes an overexpression of the p16 protein on chromosome 9p21, the mutation of which is associated with OSCCs (Otha, 2009). Furthermore, E7 blocks cell apoptosis by inhibiting the keratinocyte Bax gene [15] and by binding to the tumor necrosis factor receptor-1 (TNF-R1) [17], leading to an increase in DNA mutations in host cells and promoting carcinogenesis.

E6 is also able to inhibit the activity of p73 [15]. Mutated cells in the basal layer then transfer the genetic mutations to daughter cells. Clinically detectable lesions are the result of uncontrolled cell growth [15].

Finally, the E6 and E7 proteins can also influence epigenetic mechanisms by inducing DNA methylation [18].

Three types of vaccines against HPV infection are presently available: Gardisil (Quadrivalent; Merck & Co., Kenilworth, NJ, USA) against HPV-6, -11, -16, and 18 [19]; Gardisil9 (Nonavalent; Merck & Co., Kenilworth, NJ, USA) against HPV-6, -11, -16, -18, -31, -33, -45, -52, and -58 [19]; and Cervarix (Bivalent; GSK, Brentford, UK) against HPV-16 and -18 [19]. HPV vaccination has reduced the incidence of cervical cancer by 68–86% among vaccinated women aged 16–19 years [20]. However, vaccination coverage rates among young adults remain low, as only 54% of women and 27% of men aged 18–26 years started vaccination in 2018, and only 22% completed it [21].

The WHO reports that only 15% of the eligible female population worldwide received the last HPV dose in 2019, which is particularly concerning in low- and middle-income countries where uterine cancer is most prevalent [21]. The COVID-19 pandemic has further impacted vaccination rates, with a 75% decline in vaccination rates among U.S. adolescents aged 13–17 years in 2020 compared with 2019, and populations with limited access to health care reported a higher likelihood of pandemic-related HPV vaccination interruption [22,23,24] (Table 1).

Altogether, since 2006, when the first HPV vaccine was introduced in the United States, 71% of adolescents aged 13–17 years have been vaccinated [21]. Although the HPV vaccine is highly effective in preventing infections caused by oral HPV genotypes 16 and 18 for at least ten years after the administration [25,26], the Food and Drug Administration has not given official approval for the use of HPV vaccination as a preventive measure for oropharyngeal cancer. As a result, this lack of endorsement has led to limited awareness regarding the beneficial correlation between HPV vaccination and oral squamous cell carcinoma incidence reduction. Nevertheless, the American Cancer Society recommends routine HPV vaccination between ages 9 and 12 years, regardless of HPV status, to be most effective in preventing HPV cancer compared to the older age [27].

Oral and dental healthcare providers have always focused primarily on the secondary prevention of HPV-related benign lesions and oral/oropharyngeal cancer through clinical examinations [10,28]. However, recent proposals by the American Dental Association (ADA) and the American Academy of Pediatric Dentistry (AAPD) suggest expanding the role of oral healthcare providers to include improving the education and awareness of HPV-related oral lesions and recommending HPV vaccination [29,30]. Accordingly, to limit viral transmission, health care workers, including dentists, should educate their patients about HPV transmission routes, virus-related skin, mucosal, and oral lesions, as well as the possibility of latent or persistent HPV infection, especially when caused by high-risk viral genotypes [3]. In addition, safe practices such as the use of condom and barriers during orogenital intercourse should be promoted as part of sexual education, and healthy lifestyles should also be promoted by supporting smoking cessation since tobacco smoking is significantly associated with HPV infection, and therefore cessation is critical for the prevention of HPV-related diseases [31,32,33,34]. Furthermore, healthcare workers, including dentists and pedodontists, should encourage vaccination as early as 9 to 10 years of age, as vaccine administration at an older age is less effective in reducing cancer risk [27].

## Figures and Tables

**Table 1:** WHO dashboard: HPV vaccination coverage by age 15, last dose, males (M) and females (F), in WHO regions and globally.

	America		Europe		Africa		South-East Asia Region		Western Pacific Region		Global	
	F	M	F	M	F	M	F	M	F	M	F	M
2021	62%	29%	28%	4%	18%		1%		7%	1%	15%	4%
2020	61%	26%	27%	2%	17%		0%		5%	1%	14%	4%
2019	59%	23%	27%	2%	13%		0%		5%	1%	13%	3%
2018	55%	18%	26%	1%	1%		0%		4%	1%	10%	2%
